# Dose-response technique combined with stable isotope tracing for drug metabolite profiling by using high-resolution mass spectrometry

**DOI:** 10.3389/fphar.2023.1293540

**Published:** 2023-12-13

**Authors:** I-Shou Lin, Chia-Ying Anderin Chuang, Chia-Lung Shih

**Affiliations:** ^1^ Department of Anesthesiology, Ditmanson Medical Foundation Chia-Yi Christian Hospital, Chia-Yi City, Taiwan; ^2^ Research Center for Environmental Changes, Academia Sinica, Taipei, Taiwan; ^3^ Clinical Research Center, Ditmanson Medical Foundation Chia-Yi Christian Hospital, Chia-Yi City, Taiwan

**Keywords:** dose-response relationship, metabolomics, time-course experiment, rosiglitazone, stable isotope tracing

## Abstract

**Background:** Mass spectrometry metabolomics-based data-processing approaches have been developed for drug metabolite profiling. However, existing approaches cannot be used to comprehensively identify drug metabolites with high efficacy.

**Methods:** Herein, we propose a two-stage data-processing approach for effective and comprehensive drug metabolite identification. The approach combines dose-response experiments with stable isotope tracing (SIT). Rosiglitazone (ROS), commonly used to treat type 2 diabetes, was employed as a model drug.

**Results:** In the first stage of data processing, 1,071 features exhibited a dose-response relationship among 22,597 features investigated. In the second stage, these 1,071 features were screened for isotope pairs, and 200 features with isotope pairs were identified. In time-course experiments, a large proportion of the identified features (69.5%: 137 out of 200 features) were confirmed to be possible ROS metabolites. We compared the validated features identified using our approach with those identified using a previously reported approach [the mass defect filter (MDF) combined with SIT] and discovered that most of the validated features (37 out of 42) identified using the MDF-SIT combination were also successfully identified using our approach. Of the 143 validated features identified by both approaches, 74 had a proposed structure of an ROS-structure-related metabolite; the other 34 features that contained a specific fragment of ROS metabolites were considered possible ROS metabolites. Interestingly, numerous ROS-structure-related metabolites were identified in this study, most of which were novel.

**Conclusion:** The results reveal that the proposed approach can effectively and comprehensively identify ROS metabolites.

## Introduction

Approaches to the processing of mass spectrometry (MS)-based metabolomics data have been developed for drug metabolite profiling (Xiao et al., 2020). These approaches can be used to identify not only common metabolites but also uncommon metabolites that cannot be predicted by analyzing known biotransformation reactions. The metabolomics data processing developed for screening drug metabolites include XCMS Online (Gowda et al., 2014), the mass defect filter (MDF) (Zhang et al., 2009), and stable isotope tracing (SIT) (Lin et al., 2010).

Existing metabolomics-based data-processing approaches have several drawbacks. XCMS Online can be used to identify drug metabolites by comparing the differences in peak abundance between two dose groups (Gowda et al., 2014), although the vast majority of identified candidates are nondrug metabolites (approximately 90%) (Hsu et al., 2016). The MDF used for metabolite identification is based on known biotransformation reactions; in these reactions, metabolite mass defects are usually discovered within a 50-mDa window, and their mass changes are almost always within a 50-Da window. When the MDF is used to identify drug metabolites, the false discovery rate is high (>90%) (Shih et al., 2018). The SIT approach was designed to trace paired peaks of isotopes, specifically native and isotope-labeled metabolites. Although the combination of statistical analysis with SIT was found to achieve a high true positive rate (>90%) when used for metabolite identification, a few structures identified (4 out of 10) could be proposed (Shih et al., 2018). Thus, a metabolomics-based data-processing approach that can comprehensively identify drug metabolites with high efficacy is needed.

Dose-response experiments have been employed in toxicant metabolite identification to determine the applicability of metabolite candidates (Hsu et al., 2011). Drug metabolites are expected to induce a dose-based response, and a dose-response experiment can thus be adopted in metabolomics-based data processing for drug metabolite identification. In a study using XCMS Online, the relative abundance of many drug metabolites was found to be significantly different between two sample groups with differing doses (Hsu et al., 2016). We therefore expected that numerous endogenous metabolites would result in a dose-based response. In addition, a two-stage data-processing approach could increase the efficacy of metabolite identification. In our previous study, the MDF was combined with SIT to improve the efficacy of drug metabolite identification compared with that of the MDF alone. This combination resulted in an improvement of the validated rate (the percentage of identified peaks indicating a dose-response relationship) from 10% to 74% (Su et al., 2022). However, the MDF-SIT combination has a major limitation when applied to drug metabolite identification; it cannot identify metabolites that fall outside mass defect windows or mass change windows. A more comprehensive approach should be developed to address this limitation. In the present study, a dose-response technique was combined with SIT for drug metabolite identification. This approach should be an effective and comprehensive means of identifying drug metabolites.

Rosiglitazone (ROS), a thiazolidinedione, is used in patients with type 2 diabetes to lower their the glucose level by reducing their insulin resistance (Camp, 2003). ROS metabolism has been investigated in rats, dogs, and humans, the major metabolites of ROS are believed to be the products of ROS N-demethylation and hydroxylation. However, ROS metabolism has not yet been investigated using high-resolution liquid chromatography (LC)-MS, which could uncover novel ROS metabolites (Su et al., 2022). The metabolism of pioglitazone (PIO), another type of thiazolidinedione, has recently been investigated using high-resolution LC-MS. Although 10 novel PIO metabolites have been identified, 9 of them are thiazolidinedione ring-opening metabolites (Su et al., 2022). However, this type of metabolite has not been identified as being involved in ROS metabolism (Bolton et al., 1996; Cox et al., 2000). The numbers of cases of myocardial infarction (Alemán-González-Duhart et al., 2016) and hepatotoxicity (Al-Salman et al., 2000; Forman et al., 2000) in patients receiving ROS have been reported to be significantly increasing. ROS may produce metabolites associated with myocardial infarction and hepatotoxicity, and further investigation into ROS metabolism is thus warranted.

This study aimed to develop a metabolomics-based data-processing approach for the investigation of drug metabolism; a two-stage approach was considered in which a dose-response experiment was combined with SIT. We expect that the developed approach can effectively and comprehensively identify drug metabolites.

## Material and methods

### Standards and reagents

The chemicals and reagents used in our experiment were obtained from the following sources. PIO (purity = 97%) standard was obtained from Toronto Research Chemicals (North York, Ontario, Canada). ROS labeled with deuterium (on the benzene ring; ROS-D_4_, purity = 96%) standard was obtained from BDG Synthesis Limited (Wellington, New Zealand). Human liver S9 fractions (20 mg/mL protein base) were obtained from Thermo Fisher Scientific (Runcorn, United Kingdom). ROS (purity ≥98%) standard, glucose-6-phosphate dehydrogenase (activity: 225 units/mg), MgCl_2_ (purity ≥98%), sodium phosphate monobasic monohydrate (purity ≥98%), sulfatase (activity: 11 units/mL), D-glucose 6-phosphate disodium salt hydrate (purity ≥98%), β-glucuronidase (activity >85,000 units/mL), β-nicotinamide adenine dinucleotide phosphate sodium salt hydrate (NADP, purity ≥98%), acetic acid (purity ≥99%), and sodium phosphate dibasic (purity ≥99%) were obtained from Sigma-Aldrich (St. Louis, MO, United States).

### Human liver incubation

To simulate the metabolism of ROS in the human liver, ROS was incubated with human liver S9 fractions. A 0.5 mL incubation sample was prepared that comprised 1 mM NADP, 0.6 U/mL glucose 6-phosphate dehydrogenase, 3 mM MgCl_2_, 3 mM glucose 6-phosphate, 3.75 mg/mL human liver S9 fraction, and 0.5 μg/mL parent drugs (ROS and ROS-D_4_) in phosphate buffer (100 mM and pH 7.4). The mixture was incubated at 37°C for 24 h. Subsequently, 13 μL of β-glucuronidase and 5 μL of sulfatase were added, after which the mixture was incubated for 90 min incubation at 37°C. Then, 28 μL of acetic acid at 20% (v/v) strength was added to the mixture to stop the enzyme reactions; next, 10 μL of PIO (1,000 ppm) was added to the mixture to normalize the peak abundances obtained through LC-MS analysis. A supernatant was obtained from the mixture through centrifugation at 13,500 rpm for 10 min, and then filtered using a polyvinylidene difluoride membrane (0.22 μm, MSonline Scientific Co., Ltd. Taiwan). The analytes in the filtered sample were extracted in methanol and were purified through solid-phase extraction performed using a C18 cartridge (Sep-Pak C18 1cc Vac Cartridge, 50 mg sorbent per cartridge, 55–105 μm); the cartridge was first preconditioned with 2 mL of acetic acid (1% v/v) and 2 mL of methanol. Finally, 1 mL of methanol was used for elution of the analytes.

### LC-MS and LC-tandem MS (MS/MS)

Incubation samples were fully scanned using the UltiMate 3000 HPLC system coupled with an Orbitrap Fusion Lumos Tribrid mass spectrometer (Thermo Fisher Scientific, San Jose, CA, United States). A total of 5 μL the incubation sample was injected into the system. Spectra were acquired at a resolution of 120,000 with a scan range from m/z 80 to 800. Chromatographic separation was performed on an ACQUITY ultraperformance LC (UPLC) bridged ethyl hydrid C18 column (2.1 mm × 100 mm, 1.7 μm); the mobile phases were 0.1% formic acid (solvent A) and methanol mixed with 0.1% formic acid (solvent B). The flow rate was set as 300 μL/min, and the following gradient elution was employed: 100% solvent A, 0–1 min; 0%–50% solvent B, 1–1.01 min; 50%–100% solvent B, 1.01–7 min; and 100% solvent B, 7–8.5 min. Ions were detected using electrospray ionization-MS analysis in positive ion mode with an ion-spray voltage of 3,500 V. Validated features were analyzed further using LC-MS/MS analysis, the conditions for which were the same as in the LC-MS analysis. The collision energy was set as 30 and 35 eV to acquire MS/MS profiles.

### Dose-response experiment combined with SIT

**FIGURE 1:**
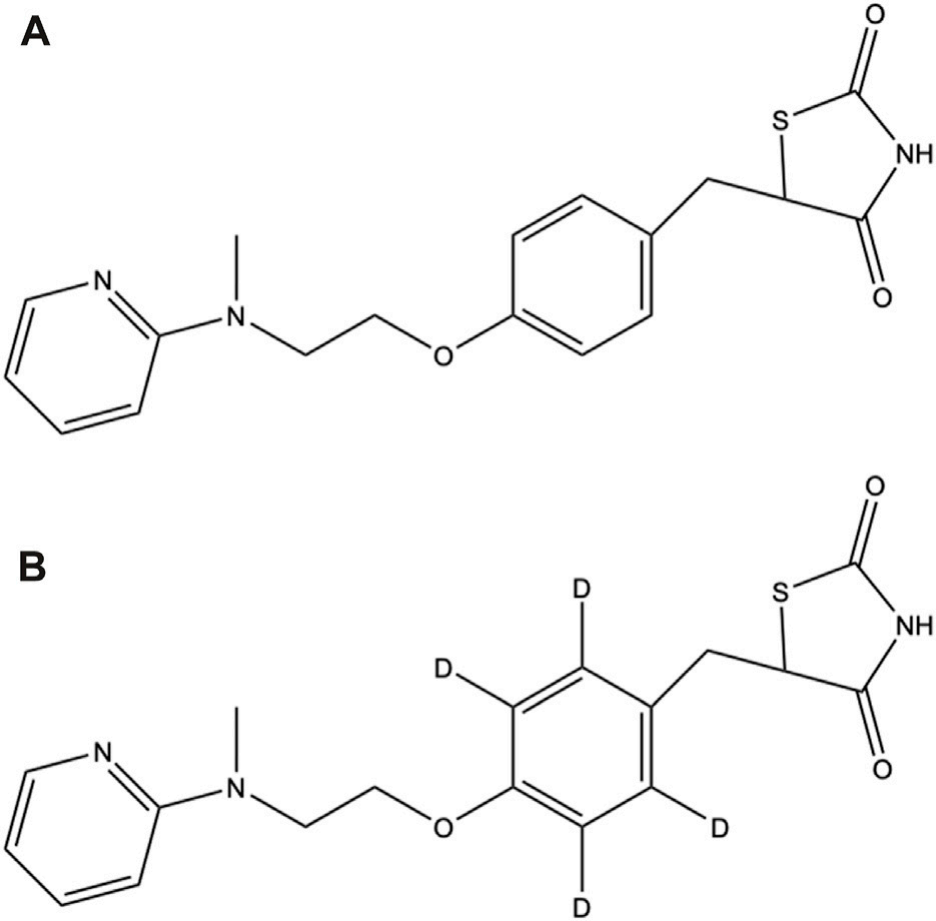
Chemical structures of **(A)** ROS and **(B)** ROS-D_4_.

The dose-response technique and SIT were conducted in a single experiment. ROS and its isotope-labeled compound (ROS-D_4_) were incubated in the same tube at one of five dose concentrations: 0, 8, 16, 32, or 64 μg/mL (n = 3 for each concentration) (Figure 1). The procedure of human enzyme incubation was the same as that described earlier. The incubation samples were analyzed using LC-MS. The Progenesis QI software (Waters, Newcastle, United Kingdom) was used to convert MS files into peak lists. We set the machine type option to “high resolution mass spectrometer”. For the alignment reference, we selected the option “Assess all runs in the experiment for suitability”. For peak picking, we set the option of the % base peak to 0, and we did not apply the option of a minimum peak width. The maximum charge was set to 20. Additionally, we did not apply the function of retention time (RT) limits. After obtaining the peak lists, we normalized the peak abundances by dividing them by the spiked PIO abundance. The normalized abundances were then used in further analysis.

### MDF-SIT combination

The metabolites identified by our current approach were compared with those identified our previously reported two-stage data-processing approach in which the MDF is combined with SIT (Su et al., 2022). ROS and its isotope-labeled compound (ROS-D_4_) were incubated in this experiment. We followed the procedure stated for the human enzyme incubation described in a previous report. One concentration (64 μg/mL) of ROS and ROS-D_4_ was adopted for the liver enzyme incubation, and three replicates were prepared.

### Time-course experiment

To validate the identified peaks as possible ROS metabolites, a time-course experiment was conducted. We again followed the procedure for human enzyme incubation procedure described previously. ROS (100 μg/mL) was incubated, and incubation samples were collected at 0, 2, 4, and 6 h later (n = 3 for each time point). The samples were analyzed using LC-MS, and Progenesis QI software was employed to convert the MS files into peak lists. The peak abundances were normalized by dividing them by the spiked PIO abundances, and these normalized abundances were then analyzed.

### Statistical analysis and data processing

All statistical analyses and data processing were performed using R software (version 4.1.0, http://r-project.org/). In the first stage of our developed approach, Spearman rank correlations between normalized peak abundances and drug doses were calculated. Any feature exhibiting a dose-response relationship was considered a candidate ROS metabolite. In the second stage, SIT was performed to identify an isotope pair consisting of a native metabolite and its isotopically labeled metabolite. The pair had to meet the following criteria: 1) a mass difference of 4.025 ± 0.001 Da, 2) an RT shift within 0.1 min, and 3) a charge state of 1. For the MDF-SIT experiment, ROS metabolite candidates were identified using the MDF. Peaks within a mass defect window of 50 mDa relative to that of the parent drug (ROS or ROS-D_4_) and within a mass change window of 50 Da around the parent drug were identified; these candidates were then screened for isotope pairs by using the screening method described previously. Finally, the identified peaks indicating isotope pairs were validated through a time-course experiment in which the Spearman rank correlations between peak abundances and the incubation time were calculated. An ROS metabolite candidate was defined to have been validated when the correlation for this metabolite was found to be positive in the time-course experiment.

### Structure elucidation

The validated metabolite candidates were further investigated using several strategies to elucidate their structure. First, the possible structures of these candidates were searched for in an online database (the human metabolome database) on the basis of their *m/z* values in the positive ion mode with a mass tolerance of 5 ppm. Second, the known biotransformation routes were used to predict possible chemical structures from the parent drug (Anari et al., 2004; Zhang et al., 2009; Ma and Chowdhury, 2011). Third, if possible structures could not be identified using the aforementioned strategies, structures were manually proposed on the basis of their pattern of fragmentation and predicted fragment elemental composition. Finally, CFM-ID software was used to fragment the proposed structures *in silico* (Allen et al., 2014). We followed the guidelines to evaluate the confidence level of the structure elucidation; this confidence was categorized into five levels: level 1, confirmed structure; level 2, probable structure; level 3, tentative candidate; level 4, unequivocal molecular formula; and level 5, exact mass (Schymanski et al., 2014)

## Results

### Dose-response experiment combined with SIT

**FIGURE 2:**
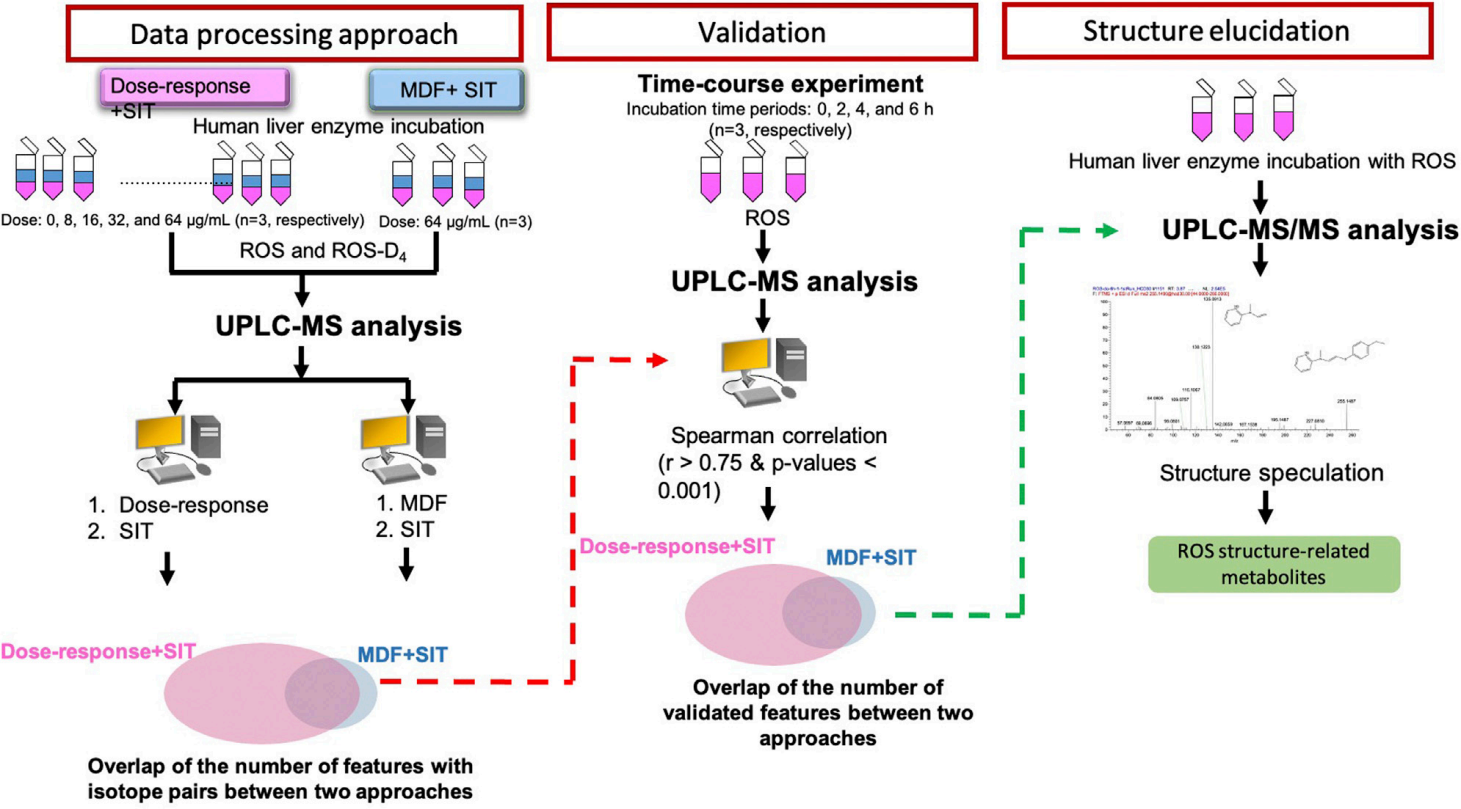
Flowchart of two-stage data-processing approaches for identifying drug metabolites by using UPLC-MS.

The flowchart of this study design is displayed in Figure 2. A two-stage data-processing approach in which a dose-response experiment was combined with SIT was developed for drug metabolite identification. MS files obtained from incubation samples were converted into peak lists (including the *m/z* value, RT, charge state, and peak abundance), and 27,323 features were identified. To remove features that could be caused by noise, any feature with mean peak abundance of lower than 1,000 in the three replicated samples with an ROS concentration of 64 μg/mL were deleted. The peak abundance of the remaining 22,597 features was normalized by dividing the abundance by the peak abundance of the spiked PIO standard.

**FIGURE 3:**
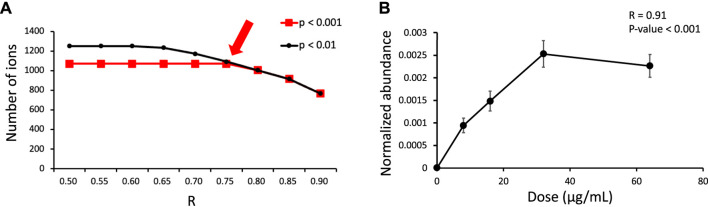
**(A)** Number of features exhibiting a dose-response relationship under different criteria (R and p obtained from Spearman correlation analysis) in the dose-response experiment. The arrow indicates the criteria that were used in this study. **(B)** Example dose-response curve for *m/z* 317.1493 at an RT of 3.13 min.

In the first stage of data processing, features with a dose-response relationship were detected. The criteria employed to determine the dose-response relationships (Figure 3A) were R > 0.75 and *p* < 0.001 (Figure 3B). These criteria were used because the number of features decreased substantially to 1,071 when these criteria were applied. In the second stage, the remaining features were screened for isotope pairs, and 200 pairs were found to meet the corresponding criteria (a mass difference = 4.025 ± 0.001 Da, RT shift <0.1 min, and charge state = 1).

The identified features with isotope pairs were validated by performing a time-course experiment. The normalized abundance and incubation time were positively correlated (R > 0.75 and *p*-value <0.001) for 139 of the 200 features. These validated features can be concluded with high confidence to be ROS metabolites.

### MDF combined with SIT

The MS files of the incubation samples were converted into peak lists, and 6,899, 6,813, and 6,895 features were obtained from the three replicated samples, respectively. To remove features that may have been caused by noise, those with a peak abundance of lower than 1,000 were deleted. This left 1,975, 1,993, and 2,119 features, respectively, which were retained for metabolite identification. MDF screening was then applied, resulting in 270, 270, and 280 features retained from the three replicates, respectively.

These features were screened for those with isotope pairs, and 66, 73, and 72 features were identified from the three replicates, respectively. Out of these features, 64 were found in at least two replicates, indicating that they were highly likely to be ROS metabolites. These features were further validated by conducting a time-course experiment. Ultimately, the normalized abundance and incubation time were discovered to be positively correlated for 41 of the 64 features.

### Chemical structure elucidation

The chemical structure of an unknown feature is a strong indication of its suitability as a drug metabolite. Synthetic standards provide the highest confidence in confirming the structure of unknown features; however, synthesizing a standard is both time-consuming and costly, especially in the present case, in which many features were identified. Elucidating the chemical structure of unknown features on the basis of MS/MS profiles is an alternative means of confirming structures with relatively high confidence (Samaraweera et al., 2020).

**FIGURE 4:**
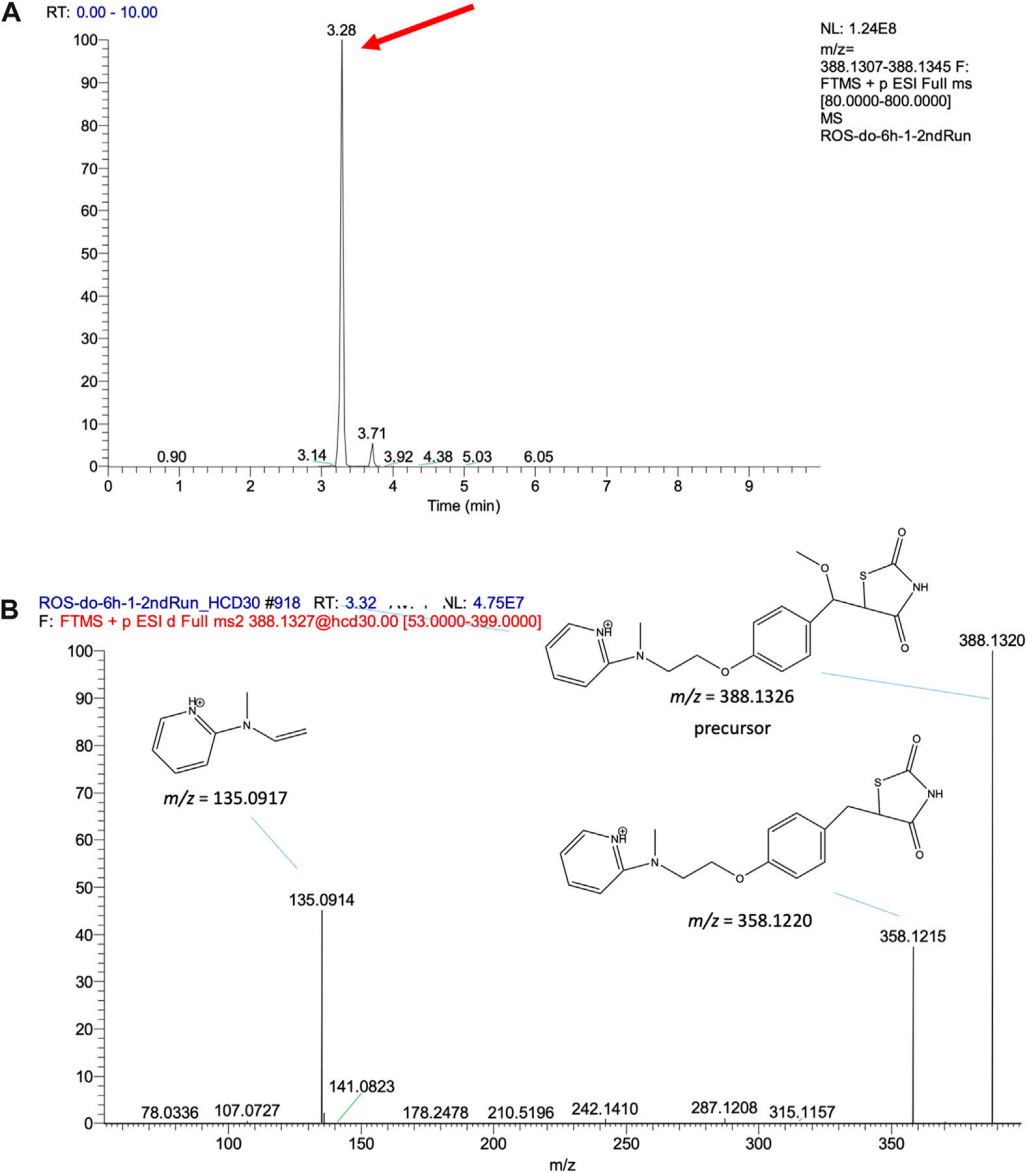
**(A)** EIC of the validated feature *m/z* 388.1326 and **(B)** its MS/MS fragment pattern and the proposed structures.

The feature at *m/z* 388.1323 is taken as an example to explain the process of structure elucidation. The extracted ion chromatogram (EIC) of the feature contained a high peak at an RT 3.28 min (Figure 4A). The identified feature was used as a processor, and its MS/MS profile was obtained through UPLC-MS/MS analysis. First, the information on the feature was searched for in the database, but no matching compounds were identified. Second, the predicated biotransformation routes from ROS were searched for the feature. One predicated biotransformation route was matched to the feature. The possible reaction was ROS hydroxylation plus methylation. The proposed structure of the feature was ROS with added OCH_2_ (Figure 4B), and CFM-ID software was used to obtain possible fragments from the structure. Two experimental fragments (*m/z* 135.0917 and 358.1215) matched the *in silico* fragments.

We carefully reviewed the EICs of the143 validated features. The EIC of one did not have a pronounced peak, and the MS/MS profiles of three could not be obtained through UPLC-MS/MS analysis; thus, the structures of these four features were not subject to further elucidation. Additionally, the precursors of 31 out of the remaining 139 features could not be found in the MS/MS profiles, and we could not confirm whether their MS/MS profiles were obtained from these features. Structure elucidation was performed for the remaining 108 features. Feature M74 was discovered to match a known compound (HMDB0060839: N-desmethyl-p-O-sulfate rosiglitazone) in the online database. This feature is a previously reported ROS metabolite (Cox et al., 2000). Features M45 and M66 could be predicted using the common biotransformation routes from ROS, and their structures could be proposed.

The chemical structures of the remaining 106 features were manually investigated and proposed. The proposed structures were fragmented using CFM-ID software, and the experimental fragments were matched to the *in silico* fragments to determine which structures we could be highly confident of; however, not all the proposed structures’ experimental fragments could be matched to the *in silico* fragments. Overall, structures could be proposed for the 74 validated features (Supplementary Figure S1); with these 74 features are considered ROS structure-related metabolites. The level of confidence in structure elucidation was assessed as being level 3 for all the 74 proposed structures. The other 34 validated features had a specific fragment (*m/z* 135.0917) that is commonly found in the MS/MS profiles of the proposed structures (Supplementary Table S1; Supplementary Figure S2). These 34 validated features may be ROS metabolites, but their structures could not be proposed in this study. Furthermore, 10 of the 34 features had unequivocal molecular formulas and were assessed with a level 4 confidence; the remaining 24 features were assessed as being level 5.

The major characteristics of the 74 ROS-structure-related metabolites are shown in Table 1. A comparison between the experimental and expected *m/z* values for these metabolites revealed high mass accuracy (mass error within 2 ppm). Most of the metabolites (70/74) had the major fragment *m/z* 135.0971 in their MS/MS profile. Some metabolites had similar *m/z* values but were eluted at different RTs; these metabolites were suspected to be isomers (such as M8 and M9). The proposed structures (n = 71) were thiazolidinedione ring-opening metabolites except for M63, M65 and M66 (Supplementary Figure S1). Of the 74 proposed structures, 26 could be identified using the MDF combined with SIT (Table 1). Of these 26 metabolites, most (n = 24) were also determined using our developed approach.

**TABLE 1:** Characteristics of the 74 structures proposed in this study.

ID	*m/z* value	RT (min)	Charge state	Specific fragment ion (*m/z* 135.0917)	Formula	Expected *m/z*	Error (ppm)^$^	Filter approach	Novel	Level of confidence^#^
M1	137.0596	3.62	1	-	C_8_H_8_O_2_	137.0598	−1.46	a	+	3
M2	243.1126	3.26	1	-	C_14_H_14_N_2_O_2_	243.1129	−1.23	a	+	3
M3	245.1283	3.15	1	+	C_14_H_16_N_2_O_2_	245.1285	−0.82	a	+	3
M4	255.1490	3.81	1	+	C_14_H_18_N_2_O	255.1492	−0.78	a	+	3
M5	255.1490	3.92	1	+	C_14_H_18_N_2_O	255.1492	−0.78	a	+	3
M6	257.1283	3.30	1	+	C_15_H_16_N_2_O_2_	257.1285	−0.78	a	+	3
M7	259.1439	3.19	1	+	C_15_H_18_N_2_O_2_	259.1442	−1.16	a	+	3
M8	271.1439	0.83	1	+	C_16_H_18_N_2_O_2_	271.1442	−1.11	a	+	3
M9	271.1439	2.95	1	+	C_16_H_18_N_2_O_2_	271.1442	−1.11	a	+	3
M10	271.1439	3.36	1	+	C_16_H_18_N_2_O_2_	271.1442	−1.11	a	+	3
M11	272.1391	3.08	1	+	C_15_H_17_N_3_O_2_	272.1394	−1.10	a	+	3
M12	273.1232	3.26	1	+	C_15_H_16_N_2_O_3_	273.1234	−0.73	a	+	3
M13	283.1439	3.18	1	+	C_17_H_18_N_2_O_2_	283.1442	−1.06	a	+	3
M14	286.1548	3.13	1	+	C_16_H_19_N_3_O_2_	286.1551	−1.05	a	+	3
M15	287.1388	3.61	1	+	C_16_H_18_N_2_O_3_	287.1391	−1.04	a	+	3
M16	287.1388	3.32	1	+	C_16_H_18_N_2_O_3_	287.1391	−1.04	a	+	3
M17	297.1595	3.29	1	+	C_18_H_20_N_2_O_2_	297.1598	−1.01	a	+	3
M18	297.1595	3.51	1	+	C_18_H_20_N_2_O_2_	297.1598	−1.01	a	+	3
M19	299.1388	3.26	1	+	C_17_H_18_N_2_O_3_	299.1391	−1.00	a	+	3
M20	299.1388	3.38	1	+	C_17_H_18_N_2_O_3_	299.1391	−1.00	a	+	3
M21	299.1388	3.48	1	+	C_17_H_18_N_2_O_3_	299.1391	−1.00	a	+	3
M22	301.1181	3.06	1	+	C_16_H_16_N_2_O_4_	301.1183	−0.66	a	+	3
M23	301.1181	0.86	1	+	C_16_H_16_N_2_O_4_	301.1183	−0.66	a	+	3
M24	301.1545	3.44	1	+	C_17_H_20_N_2_O_3_	301.1547	−0.66	a	+	3
M25	301.1545	3.20	1	+	C_17_H_20_N_2_O_3_	301.1547	−0.66	a	+	3
M26	302.1497	3.00	1	+	C_16_H_19_N_3_O_3_	302.1500	−0.99	a	+	3
M27	303.1337	0.85	1	+	C_16_H_18_N_2_O_4_	303.1340	−0.99	a	+	3
M28	303.1337	3.06	1	+	C_16_H_18_N_2_O_4_	303.1340	−0.99	a	+	3
M29	312.1704	4.07	1	+	C_18_H_21_N_3_O_3_	312.1707	−0.96	a	+	3
M30	312.1704	3.33	1	+	C_18_H_21_N_3_O_3_	312.1707	−0.96	a and b	+	3
M31	313.1544	3.27	1	+	C_18_H_20_N_2_O_3_	313.1547	−0.96	a and b	+	3
M32	317.1493	3.13	1	+	C_17_H_20_N_2_O_4_	317.1496	−0.95	a and b	+	3
M33	317.1493	3.23	1	+	C_17_H_20_N_2_O_4_	317.1496	−0.95	a and b	+	3
M34	317.1494	0.87	1	+	C_17_H_20_N_2_O_4_	317.1496	−0.63	a and b	+	3
M35	326.1497	3.24	1	+	C_18_H_19_N_3_O_3_	326.1500	−0.92	a and b	+	3
M36	327.1337	3.19	1	+	C_18_H_18_N_2_O_4_	327.1340	−0.92	a and b	+	3
M37	327.1337	3.36	1	+	C_18_H_18_N_2_O_4_	327.1340	−0.92	a and b	+	3
M38	329.1493	3.40	1	+	C_18_H_20_N_2_O_4_	329.1496	−0.91	a	+	3
M39	338.1499	3.51	1	+	C_19_H_19_N_3_O_3_	338.1500	−0.30	a	+	3
M40	339.1337	3.54	1	+	C_19_H_18_N_2_O_4_	339.1340	−0.88	a and b	+	3
M41	340.1290	3.46	1	+	C_18_H_17_N_3_O_4_	340.1292	−0.59	a and b	+	3
M42	341.1493	3.38	1	+	C_19_H_20_N_2_O_4_	341.1496	−0.88	a	+	3
M43	343.1285	3.17	1	+	C_18_H_18_N_2_O_5_	343.1289	−1.17	a	+	3
M44	343.1286	3.24	1	+	C_18_H_18_N_2_O_5_	343.1289	−0.87	a	+	3
M45	344.1426	3.46	1	+	C_18_H_21_N_3_O_2_S	344.1428	−0.58	a and b	+	3
M46	345.1265	3.54	1	+	C_18_H_20_N_2_O_3_S	345.1268	−0.87	a and b	+	3
M47	345.1442	3.08	1	+	C_18_H_20_N_2_O_5_	345.1445	−0.87	a and b	+	3
M48	345.1443	0.85	1	+	C_18_H_20_N_2_O_5_	345.1445	−0.58	a and b	+	3
M49	356.1602	3.25	1	+	C_19_H_21_N_3_O_4_	356.1605	−0.84	a	+	3
M50	359.1599	3.30	1	+	C_19_H_22_N_2_O_5_	359.1602	−0.84	a and b	+	3
M51	361.1391	3.05	1	+	C_18_H_20_N_2_O_6_	361.1395	−1.11	a	+	3
M52	363.0829	3.44	1	+	C_17_H_18_N_2_O_3_S_2_	363.0832	−0.83	a and b	+	3
M53	363.1007	3.26	1	+	C_17_H_18_N_2_O_5_S	363.1010	−0.83	a	+	3
M54	365.1162	3.15	1	+	C_17_H_20_N_2_O_5_S	365.1166	−1.10	a	+	3
M55	368.1965	3.52	1	+	C_21_H_25_N_3_O_3_	368.1969	−1.09	a	+	3
M56	372.1551	3.63	1	+	C_19_H_21_N_3_O_5_	372.1554	−0.81	a	+	3
M57	373.1391	3.21	1	+	C_19_H_20_N_2_O_6_	373.1395	−1.07	a and b	+	3
M58	373.1755	3.50	1	+	C_20_H_24_N_2_O_5_	373.1758	−0.80	a	+	3
M59	375.1370	3.52	1	+	C_19_H_22_N_2_O_4_S	375.1374	−1.07	a and b	+	3
M60	375.1548	3.11	1	+	C_19_H_22_N_2_O_6_	375.1551	−0.80	a and b	+	3
M61	377.1162	3.24	1	+	C_18_H_20_N_2_O_5_S	377.1162	0.00	a and b	+	3
M62	381.1112	3.11	1	+	C_17_H_20_N_2_O_6_S	381.1115	−0.79	b	+	3
M63	384.1009	3.74	1	+	C_19_H_17_N_3_O_4_S	384.1013	−1.04	a	+	3
M64	384.1551	3.27	1	+	C_20_H_21_N_3_O_5_	384.1554	−0.78	a and b	+	3
M65	385.1690	3.42	1	+	C_20_H_24_N_4_O_2_S	385.1693	−0.78	a	+	3
M66	388.1323	3.30	1	+	C_19_H_21_N_3_O_4_S	388.1326	−0.77	a and b	+	3
M67	391.1319	3.41	1	+	C_19_H_22_N_2_O_5_S	391.1323	−1.02	b	+	3
M68	393.1919	3.38	1	+	C_22_H_24_N_4_O_3_	393.1922	−0.76	a	+	3
M69	397.0706	3.61	1	+	C_17_H_20_N_2_O_3_S_3_	397.0709	−0.76	a	+	3
M70	403.1319	3.74	1	+	C_20_H_22_N_2_O_5_S	403.1323	−0.99	a and b	+	3
M71	405.1476	3.61	1	+	C_20_H_24_N_2_O_5_S	405.1479	−0.74	a and b	+	3
M72	406.1969	3.02	1	+	C_20_H_27_N_3_O_6_	406.1973	−0.98	a	+	3
M73	406.1970	0.84	1	+	C_20_H_27_N_3_O_6_	406.1973	−0.74	a	+	3
M74	440.0586	4.58	1	+	C_17_H_17_N_3_O_7_S_2_	440.0581	1.14	a	-	3

RT, retention time; $, mass error between experimental and expected m/z values. a, proposed approach; b, MDF-SIT combination; #, confidence level of structure elucidation.

## Discussion

In this study, we developed a metabolomics-based data-processing approach for comprehensively and effectively identifying drug metabolites. The proposed approach involves a two stages: a dose-response experiment and SIT. The results of a time-course experiment indicated that a high proportion of the identified features (69.5%) were confirmed to be possible ROS metabolites. Moreover, numerous ROS-structure-related metabolites were identified using our developed approach, and most of the metabolites identified using the MDF-SIT combination were also identified by using our developed approach. The findings indicate that our proposed approach is superior to the MDF-SIT combination for drug metabolite identification.

The MDF used a mass defect window relative to the parent drug for screening possible drug metabolites (Zhang et al., 2009); nevertheless, many interference features are identified by the MDF, and the efficacy of its metabolite identification is thus extremely low. To overcome this disadvantage, we previously developed a two-stage data-processing approach in which the MDF is combined with SIT and used for PIO metabolite identification (Su et al., 2022); the results demonstrated that the validated rate of identified features improved significantly from 10% to 74% (Su et al., 2022). In the present study, we adopted our approach for ROS metabolite identification and again obtained a high validation rate (64%). The difference in validation rate could have resulted from the different approaches used for metabolite validation. Dose-response experiments were employed in our previous study, whereas time-course experiments were used in this study. The present results confirm that the two-stage data-processing approach effectively increases the efficacy of metabolite identification.

The MDF is widely used to identify drug metabolites. Although the efficacy of metabolite identification can be improved by combining the MDF with SIT, some metabolites out of the mass defect shift window (50 mDa) or mass window (50 Da) relative to the parent drug cannot be identified. To overcome the limitations of the MDF, a new approach for comprehensively identifying drug metabolites is needed. Dose-response experiments were adopted previously to validate toxicant metabolites (Hsu et al., 2011) but have not yet been used for identifying drug metabolites. Thus, we attempted to combine a dose-response experiment with SIT for the purpose of effectively and comprehensively identifying drug metabolites.

The first stage of our developed approach revealed that a dose-response relationship existed for 1,071 features (Figure 3B), but only 200 features had isotopic pairs. This suggests that many endogenous metabolites created during human liver enzyme incubation can also exhibit a dose-response relationship. Incorporating SIT into our approach considerably reduced the number of these endogenous metabolites (871 features). Furthermore, 139 out of the 200 features (69.5%) were confirmed to be possible ROS metabolites in the time-course experiment. A relatively high validation rate of 69.5% was obtained using our developed approach; the equivalent rate obtained when using the MDF-SIT combination was 74% (Su et al., 2022).

**FIGURE 5:**
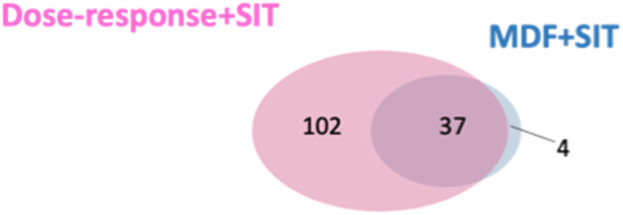
Overlap between validated ROS metabolite ions identified in dose-response experiments combined with SIT and the MDF combined with SIT.

We compared the validated features identified using our developed approach and identified through the MDF combined with SIT. More validated features were identified through our developed approach (n = 139) than those identified using the MDF-SIT combination (n = 41). Moreover, 37 out of the 41 validated features obtained through the MDF-SIT combination were also detected using our proposed approach (Figure 5). These results demonstrate that the proposed approach is a more effective and comprehensive way of identifying drug metabolites compared with the MDF-SIT combination.

ROS metabolites have been detected in human urine, and 15 ROS metabolites have been identified (Cox et al., 2000), with several of these 15 being sulfate- or glucuronide-conjugated metabolites. Except for M74, no conjugated metabolites were discovered in our study because deconjugation was performed in our incubation samples. The remaining reported ROS metabolites have 5 *m/z* values (*m/z* 374.1166, 344.1061, 282.0431, 276.0798, and 360.1013). A comparison of the previously reported ROS metabolites with those identified in this study revealed that none of the aforementioned 5 *m/z* values were found in the 74 ROS-structure-related metabolites (Table 1). We searched for these 5 *m/z* values in our peak lists, and two of them (*m/z* 374.1166 and 344.1061) were found. The feature with *m/z* 374.1166 at an RT of 3.29 min exhibited a dose-response relationship, but its isotopic labeled metabolite (*m/z* 378.1416) was not detected. The feature with *m/z* 344.1061 did not exhibit a dose-response relationship and therefore could not be identified using our approach. We also manually searched for the previously reported metabolites in the MS files. We could not identify another set of previously reported ROS metabolites (*m/z* 282.0431, 276.0798, and 360.1013) in our MS files from either the time-course experiment or the dose-response experiment. The two experiments involved different incubation times: 24 h and a maximum of 6 h for the dose-response experiment and time-course experiment, respectively. Therefore, the incubation time did not seem to affect the generation of ROS metabolites.

Numerous possible ROS metabolites were identified in this study, including 74 ROS-structure-related and 34 possible ROS metabolites. Recent advancements in high-resolution UPLC-MS instrumentation, along with the utilization of an untargeted metabolomics-based data processing approach, have led to a larger number of ROS metabolites being identified in this study than in a previous study (n = 15) (Cox et al., 2000). PIO, another thiazolidinedione drug, had structure similar to that of ROS. Therefore, the number of ROS metabolites should be similar to that of PIO metabolites. In our previous study, we used the same UPLC-MS system but different metabolomics-based data processing approach (a time-course experiment combined with SIT) for PIO metabolite identification (Ting et al., 2023). However, that study identified a relatively small number of PIO metabolites (n = 20) (Ting et al., 2023). In the present study, we conducted time-course experiments to validate the identified features, and many features were validated. This indicates that ROS metabolism produces a large number of metabolites.

Thiazolidinedione ring-opening metabolites have been identified in metabolism of PIO (another type of thiazolidinedione drug) (Campos et al., 2018). However, these types of metabolites were not identified in ROS metabolism in a previous study (Cox et al., 2000). In the present study, we discovered that those thiazolidinedione ring-opening metabolites could also be identified in ROS metabolism, with 71 possible thiazolidinedione ring-opening metabolites determined. These ring-opening metabolites were also found in other thiazolidinedione drugs (Alvarez-Sánchez et al., 2006). Thus, thiazolidinedione ring-opening metabolites are also expected to exist in ROS metabolism. Therefore, our proposed structures, which include a considerable number of thiazolidinedione ring-opening metabolites, are reliable.

This study has several limitations in this study. Although our approach identified numerous novel ROS metabolites, only one previously reported ROS metabolite was identified. Most of the previously reported ROS metabolites were not detectable by our UPLC-MS system and therefore could not be identified using our developed approach. Using different sample species and different UPLC-MS systems may result in an inability to detect certain ROS metabolites. Furthermore, 74 ROS metabolite candidates were identified, and their structures could be proposed; however, these structures could not be confirmed in this study. To establish the validity of these structures, synthetic standards must be used. Moreover, 34 features were possible ROS metabolites because they exhibited a specific fragment observed in previously reported ROS metabolites. However, their structures could not be determined in this study. In the future, an optimal approach for chemical structure elucidation should be developed. Finally, only one drug was investigated in this study. To confirm the effectiveness and comprehensiveness of our approach in identifying drug metabolites, other types of drugs should be investigated in future studies. Overall, our proposed approach has some limitations, but we believe that its benefits far outweigh its potential drawbacks.

In conclusion, our two-stage metabolomics-based data-processing approach is a more effective and comprehensive way of identifying drug metabolites compared with the MDF-SIT combination. Our results showed that the approach identified ROS metabolites with a high validation rate, and could identify most of the metabolites identified by the MDF. Moreover, many ROS metabolites that the MDF did not identify could be identified by our proposed approach. The developed approach is a simple and effective way of comprehensively identifying ROS metabolites and offers a new avenue for drug metabolite identification. To confirm the effectiveness and comprehensiveness of our approach, it should be validated by using other types of drugs or comparing it with alternative approaches.

## Data Availability

The raw data supporting the conclusion of this article will be made available by the authors, without undue reservation.
