# Supplementary material for: Dose-response technique combined with stable isotope tracing for drug metabolite profiling by using high-resolution mass spectrometry
Source: Front Pharmacol. 2023 Dec 13;14:1293540. doi: 10.3389/fphar.2023.1293540 (PMC10753831; doi:10.3389/fphar.2023.1293540)
Supplement: Supplementary file 1 [file Table2.DOCX]

Figure S1. MS/MS profiles of the 74 identified ROS metabolite ions and the proposed structures.


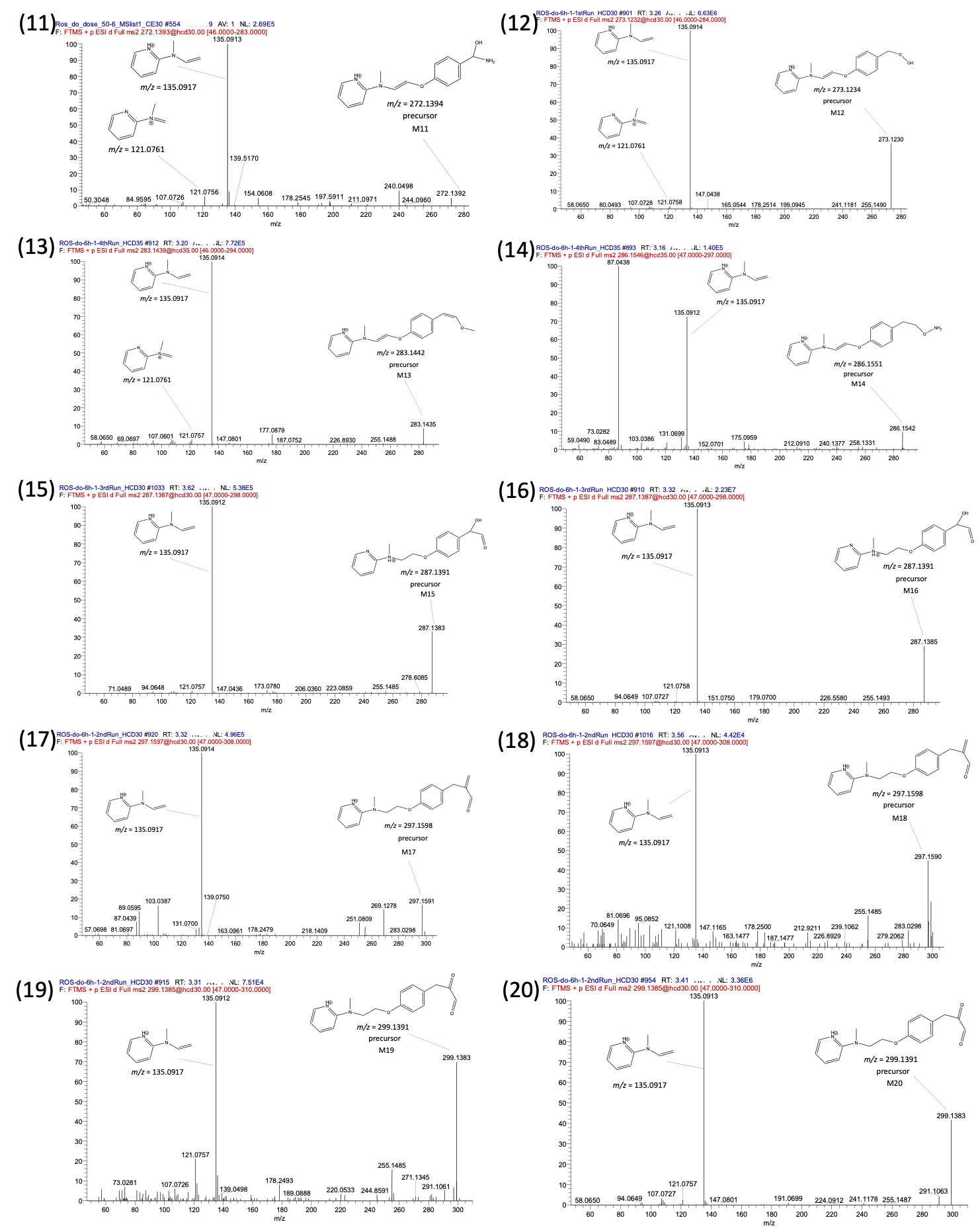


Figure S1. Continued.

Figure S1. Continued.

Figure S1. Continued.

Figure S1. Continued.

Figure S1. Continued.

Figure S1. Continued.

Figure S1. Continued.

Figure S2. MS/MS profiles of the identified 34 possible ROS metabolite ions.

Figure S2. Continued.

Figure S2. Continued.

Figure S2. Continued.

Table S1. Major characteristics of the 34 identified ROS metabolite ions.

| ID | *m/z* value | RT (min) | Charge state | Specific fragment ion (135.0917) | Formula | Expected m/z | Error (ppm) | Filter approach | Level of confidence# |
| --- | --- | --- | --- | --- | --- | --- | --- | --- | --- |
| M74 | 373.1689 | 0.83 | 1 | + |  |  |  | a | 5 |
| M75 | 373.1690 | 3.03 | 1 | + |  |  |  | a and b | 5 |
| M76 | 377.0985 | 3.74 | 1 | + |  |  |  | a | 5 |
| M77 | 377.0985 | 4.05 | 1 | + |  |  |  | a | 5 |
| M78 | 383.1057 | 3.56 | 1 | + |  |  |  | a and b | 5 |
| M79 | 408.0865 | 4.07 | 1 | + |  |  |  | a and b | 5 |
| M80 | 409.1247 | 4.02 | 1 | + |  |  |  | a | 5 |
| M81 | 416.1635 | 3.51 | 1 | + | C_21_H_25_N_3_O_4_S | 416.1639 | -0.96 | a | 4 |
| M82 | 419.1632 | 3.77 | 1 | + | C_21_H_26_N2O_5_S | 419.1636 | -0.95 | a | 4 |
| M83 | 419.1809 | 3.11 | 1 | + | C_21_H_26_N_2_O_7_ | 419.1813 | -0.95 | a | 4 |
| M84 | 421.0883 | 3.51 | 1 | + |  |  |  | a | 5 |
| M85 | 423.1040 | 3.63 | 1 | + |  |  |  | a | 5 |
| M86 | 444.1584 | 3.58 | 1 | + | C_22_H_25_N_3_O_5_S | 444.1588 | -0.90 | a | 4 |
| M87 | 449.1738 | 3.65 | 1 | + | C_22_H_28_N_2_O_6_S | 449.1741 | -0.67 | a | 4 |
| M88 | 451.1352 | 4.19 | 1 | + | C_21_H_26_N_2_O_5_S_2_ | 451.1356 | -1.11 | a | 4 |
| M89 | 458.1918 | 3.13 | 1 | + | C_23_H_27_N_3_O_7_ | 458.1922 | -0.87 | a | 4 |
| M90 | 463.1721 | 3.11 | 1 | + | C_22_H_26_N_2_O_9_ | 463.1711 | 2.16 | a | 4 |
| M91 | 472.0306 | 5.13 | 1 | + |  |  |  | a | 5 |
| M92 | 479.1669 | 3.15 | 1 | + | C_23_H_30_N_2_O_5_S | 479.1669 | 0.00 | a | 4 |
| M93 | 480.1254 | 3.46 | 1 | + | C_21_H_25_N_3_O_6_S_2_ | 480.1258 | -0.83 | a | 4 |
| M94 | 494.1410 | 3.51 | 1 | + |  |  |  | a | 5 |
| M95 | 495.1363 | 3.41 | 1 | + |  |  |  | a | 5 |
| M96 | 499.0093 | 3.61 | 1 | + |  |  |  | a | 5 |
| M97 | 526.1129 | 3.71 | 1 | + |  |  |  | a | 5 |
| M98 | 527.1300 | 3.80 | 1 | + |  |  |  | a | 5 |
| M99 | 527.1301 | 3.94 | 1 | + |  |  |  | a | 5 |
| M100 | 527.1301 | 4.07 | 1 | + |  |  |  | a | 5 |
| M101 | 541.1304 | 3.38 | 1 | + |  |  |  | a | 5 |
| M102 | 541.1458 | 4.55 | 1 | + | C_27_H_28_N_2_O_6_S_2_ | 541.1467 | -1.66 | a | 4 |
| M103 | 559.1020 | 4.18 | 1 | + |  |  |  | a | 5 |
| M104 | 559.1021 | 4.02 | 1 | + |  |  |  | a | 5 |
| M105 | 661.2141 | 3.61 | 1 | + |  |  |  | a | 5 |
| M106 | 663.2299 | 3.76 | 1 | + |  |  |  | a | 5 |
| M107 | 695.2021 | 3.92 | 1 | + |  |  |  | a | 5 |

RT: retention time. error: mass difference between experimental and expected m/z values; a: our developed approach; b: mass defect filter and stable isotope tracing; #: confidence level of structure elucidation.
